# In-field and in-vitro study of the moss *Leptodictyum riparium* as bioindicator of toxic metal pollution in the aquatic environment: Ultrastructural damage, oxidative stress and HSP70 induction

**DOI:** 10.1371/journal.pone.0195717

**Published:** 2018-04-12

**Authors:** Sergio Esposito, Stefano Loppi, Fabrizio Monaci, Luca Paoli, Andrea Vannini, Sergio Sorbo, Viviana Maresca, Lina Fusaro, Elham Asadi karam, Marco Lentini, Alessia De Lillo, Barbara Conte, Piergiorgio Cianciullo, Adriana Basile

**Affiliations:** 1 Dipartimento di Biologia, University of Naples Federico II, Complesso Univ. Monte Sant’Angelo, Napoli, Italy; 2 Dipartimento di Scienze della Vita, University of Siena, Siena, Italy; 3 Ce.S.M.A, Section of Microscopy, University of Naples Federico II, Complesso Univ. Monte Sant’Angelo, Napoli, Italy; 4 Dipartimento di Biologia Ambientale, Università Sapienza, Roma, Italy; 5 Biology Department, Shahid Bahonar University of Kerman, Kerman, Iran; Institute for Sustainable Plant Protection, C.N.R., ITALY

## Abstract

This study evaluates the effects of toxic metal pollution in the highly contaminated Sarno River (South Italy), by using the aquatic moss *Leptodictyum riparium* in bags at 3 representative sites of the river. Biological effects were assessed by metal bioaccumulation, ultrastructural changes, oxidative stress, as Reactive Oxygen Species (ROS) production and Glutathione S-transferase (GST) activity, as well as Heat Shock Proteins 70 (HSP70s) induction. The results showed that *L*. *riparium* is a valuable bioindicator for toxic metal pollution of water ecosystem, accumulating different amounts of toxic metals from the aquatic environment. Toxic metal pollution caused severe ultrastructural damage, as well as increased ROS production and induction of GST and HSP70s, in the samples exposed at the polluted sites. To assess the role and the effect of toxic metals on *L*. *riparium*, were also cultured *in vitro* with Cd, Cr, Cu, Fe, Ni, Pb, Zn at the same concentrations as measured at the 3 sites. Ultrastructure, ROS, GST, and HSP70s resulted severely affected by toxic metals. Based on our findings, we confirm *L*. *riparium* as a model organism in freshwater biomonitoring surveys, and GST and HSP70s as promising biomarkers of metal toxicity.

## Introduction

The contamination of rivers is of special concern, since they may transport contaminants, among which toxic metal, to areas far removed from any local pollution source, thus posing at risk even pristine ecosystems.

Toxic metal pollution is a major concern worldwide, and the use of living organisms for monitoring these pollutants is widely accepted [1]. Whitton et al. [2] suggested the use of ten macrophytes for biomonitoring toxic metals in European rivers and streams; among them, the aquatic moss *Leptodictyum riparium* (Hedw.) Warnst (*Bryophyta*). Bioaccumulation ability, tissue localization, toxic effects of metals and antioxidant activity in this species were investigated in previous studies [3–5], confirming its suitability for biomonitoring freshwater pollution. Furthermore, *L*. *riparium* resulted the most effective among 3 freshwater plant species in bioaccumulating metals *in vitro* [4].

Among the damages induced by environmental stress, and namely by toxic metal pollution, the disruption of main metabolic pathways, primarily due to the unfolding of enzymes and proteins, represents one of the first, and most devastating, effects on living organisms. As a consequence, cells react by activating a number of defense mechanisms: the induction, synthesis and activation of chaperons represent the most evident and widespread of them [6]. Among the different groups and families of chaperons, exhibiting different functions and roles, it is widely accepted that Heat Shock Proteins 70 (HSP70s) represent the most conserved and widespread protectants of protein structures in all cells. Their role as cell protectants under pollution stress has already been demonstrated [3,7]

Various abiotic stressors lead to the overproduction of Reactive Oxygen Species (ROS) in plants. These molecular species are highly reactive and can play a dual role in plants. In fact, they can either act as toxic agents or work as signals, which then trigger and regulate biological processes, such as cell death or adaptation responses to environmental stress, or even main physiological processes, such as cell proliferation and differentiation [8–10]. As toxicants, they can cause severe damage, which ultimately results in an oxidative stress [11–13]. This can trigger redox-sensitive pathways that lead to different alterations, such as protein carbonylation, DNA damage, activation of kinase cascades and transcription factors, which ultimately affect cellular essential metabolic activities and viability. Thus, given the need of maintaining ROS concentrations within specific ranges, plants have very efficient enzymatic and non-enzymatic antioxidant machinery, able to control ROS overproduction [11–13]. Therefore, measurement of the intracellular ROS content and/or the measure of the activity of antioxidant enzymes, e.g. glutathione S-transferase (GST), are considered valuable indicators of overall changes of the intracellular redox state [14,15].

The Sarno River (Campania region, Southern Italy), is known as one of the most polluted rivers in Europe: the whole basin (25 km long, 500 km2 wide, hosting 700,000 inhabitants) has been declared as “area with high risk of environmental crisis” [16]. The heavy urbanization and industrialization of this area caused a strong impact on the health of the local population, with significant increase in cerebrum-vascular diseases, lymphoma and cancers [17].

This study aimed at proposing an experimental protocol that allows determining contribution of the different pollutants to the overall biological effect, by carefully comparing in field and *in vitro* results. To this purpose, we evaluated the biological effects of water pollution by toxic metals in one of the most polluted river in Europe using the aquatic cosmopolitan moss *L*. *riparium*, a species able to strongly accumulate toxic metals [6]. Biological effects were studied in field and *in vitro*, considering metal bioaccumulation, oxidative stress (content of ROS and GST antioxidant enzyme activity), ultrastructural damage and HSP70s induction.

## Materials and methods

*L*. *riparium* was widely present in the Mediterranean area, and in the Sarno river basin from springs to the river mouth; in order to use similar specimens for the experimental design, samples were collected at the Botanical Gardens of the University of Naples “Federico II,” Italy. Moss grew in a basin at a depth of 20–25 cm and temperature of 17–20°C. Homogeneous samples of about 2 g fresh weight (fw) were washed with deionized water and placed into 1 mm^2^ meshed nylon bags, as recommended by Kelly et al. [18]. Six bags for each site were exposed for one week during July 2013 at a water depth of 25 cm in the River Sarno (the average temperature of the water was 17.5–19.0°C). The moss bags were exposed along the Sarno river path in three sites (S1 Table) characterized by different metal concentrations, as detailed in Basile et al. [19], and featured with good, poor, and very poor water quality respectively [20].

A summary of the three selected sites are provided below:

- “Rio Foce” (site A) near the spring, before pollution sources, as control site; 40°49’56.269” N, 14°35’27.103° E. Sarno River spring is located at 30 m asl, at the slope of the Saro Mount. This represents the Western extremity of Picentini Mounts, characterized by a forest extension of over 40,000 hectares, rich in beech, maple, alder, and chestnut, and numerous streams that make the area the richest potable drinking water tank in southern Italy.
- “San Marzano sul Sarno” (site B), before the confluence with the Alveo Comune Nocerino; this site is affected mainly by urban pollution. The Alveo Comune Nocerino is originated by the confluence of Solofrana and Cavaiola, and is the main Sarno tributary; 40°46’47.971” N, 14°34’23.296” E.
- “Scafati” (site C), the heavily polluted site, after the confluence of Alveo Comune Nocerino collecting Cavaiola e Solofrana streams, affected by heavy industrial (leather tannery; agri-food) and agricultural (tomatoes; fruit trees; vineyards) wastewaters. 40°44’48.812” N, 14°31’37.653” E.

No specific permissions were required for these locations/activities because they were not necessary and we confirm that the field studies did not involve endangered or protected species.

In parallel with in-situ toxic metal exposition, a twin experiment was performed to further evaluate the effects on ultrastructure and biochemistry of metals using manipulative laboratory infrastructure. For this purpose we exposed *in vitro* the moss to the same metal concentrations water solution and in the same condition measured in the experimental 3 sites. In addition, to assess the effects of the single metals, the moss was exposed *in vitro* to single toxic metal concentrations as measured at the most polluted environment (site C). Treatments consisted in the addition to the growth medium of the metals as soluble salts: CdCl_2_, Cr(Cl)_3_, CuSO_4_, FeCl_2_, NiCl_2_, Pb(CH3COO)_2_, ZnCl_2_. In this way the studied metals were readily available to the moss.

*In vitro* cultures were done as previously reported [7]; after collection, single gametophytes were carefully cleaned and washed with deionised water and then the surface was sterilized with ethanol 70% and NaClO 2%. Then samples were then put into Petri dishes (10 cm diameter), 20 specimens per dish. The specimens were cultured with sterile modified Mohr medium, pH 7.5 [7] and in the same medium with the addition of the metal salts. The cultures were kept in a climatic room with a temperature of 13/20°C, 70% constant RH, and photoperiod of 16/8 hours of light/dark. The specimens were maintained in the growth chamber for 7 days.

Experiments on gametophyte cultures were run in triplicate and repeated three times.

### Bioaccumulation of toxic metals

After exposure along the River Sarno, moss samples were air-dried at 40°C to constant weight and then frozen in liquid nitrogen, pulverized and homogenized with a ceramic mortar and pestle. About 300 mg of moss powder was mineralized with a mixture of 6 mL of 70% HNO_3_, 0.2 mL of 60% HF and 1 mL of 30% H_2_O_2_ (ultra-pure reagent grade). Digestion of samples was carried out in a microwave digestion system (Milestone Ethos 900) for a total time of 30 min. Concentrations of selected toxic metals (Cd, Cr, Cu, Fe, Ni, Pb, Zn), expressed on a dry weight basis, were determined by ICP-MS (Perkin-Elmer Sciex 6100) on three subsamples for each site. Analytical quality was checked by analysing the Certified Reference Material BCR 61 “aquatic moss”. Precision of analysis was estimated by the coefficient of variation of 3 replicates and was found to be <10% for all elements.

### Ultrastructural observations

Leaflets on the moss stem ca. 5 mm below the apex were used for TEM observations. Samples were fixed in 3% glutaraldehyde in phosphate buffer (pH 7.2–7.4) for 2 h at room temperature and post-fixed with buffered 1% OsO_4_ for 1.5 h at room temperature, dehydrated with ethanol and propylene oxide and embedded in Spurr’s epoxy medium [21]. Ultra-thin (50 nm thick) sections were mounted on 300-mesh copper grids, then stained with uranyl acetate and lead citrate, and observed with a Philips EM 208S TEM [21].

### HSP70s analysis

*L*. *riparium* samples of 1–5 g fw were frozen and powdered in liquid nitrogen using a mortar and pestle. HSP70s were extracted in 50 mM phosphate buffer at pH 7.5 with 10% glycerol; the homogenate was then filtered through four layers of muslin and centrifuged for 20 min at 20,000g at 4°C. The supernatant fraction was designated as the crude extract and used for SDS-PAGE analysis and Western blots. SDS-PAGE was performed using 10% acrylamide resolving gel with a 4% stacking gel. Before loading, samples were boiled for 10 min, in the presence of BBF to ensure protein denaturation. Proteins were subjected to electrophoresis under a constant voltage of 180 V, 40 mA for 90 min. For Western blot analysis, the separated polypeptides were transferred from gels to a nitrocellulose membrane (Hybond, Amersham Biosciences) soon after the SDS-PAGE run, then incubated for 2 h at room temperature with antibodies raised against bovine heart HSP70 (Sigma). Western blotting on the same extracts using anti-tubulin antisera (Sigma) were made to check the equal loading of the sample lanes, as previously described [22]. The results bands were analysed with densitometric analyses using Quantity-One software (Bio-Rad) (not shown). After washing, the membranes were incubated with secondary antibodies coupled to horseradish peroxidase and polypeptides immunoreacting were revealed by enhanced chemiluminescence using the ECL Prime kit (Ge Healthcare) as described in Cardi et al. [23]. As control, the same samples were tested against anti-alfa-tubulin antibodies, to check the equal loading of the lanes. All electrophoresis and western blotting analyses were performed with in a Mini-PROTEAN Tetra cell electrophoresis chamber (Bio-Rad), equipped with EPS 301 power supply (Ge Healthcare).

### ROS content

A fluorescent technique using 2’,7’-dichlorofluorescin diacetate (DCFH-DA) has been used for quantitative measurement of ROS production. DCFH-DA is de-esterified intracellularly and turns to nonfluorescent 2’,7’-dichlorofluorescin (DCFH). DCFH is then oxidized by ROS to highly fluorescent 2′,7′-dichlorofluorescein (DCF) [24]. Briefly, leaf samples were immediately frozen in liquid nitrogen and ground thoroughly with prechilled mortar and pestle. The resulting powder (150 mg) was then resuspended in TrisHCl 40 mM pH 7.4, sonicated, and centrifuged at 12,000g for 30 min. The supernatant (500 μL) was collected and protein content determined. An aliquot (10 μL) of each sample was incubated with 5 μM DCFH-DA for 30 min at 37°C followed by recording of the final fluorescence value, which was detected at excitation (488 nm) and emission (525 nm) wavelength (SyneryTM HTX Multi-Mode). DCF formation was quantified from a standard curve (0.05–1.0 μM). The analysis were carried out on five subsamples for each site.

### Glutathione S-transferase activity

Glutathione S-transferase (GST, EC 2.5.1.18) activity was measured using a commercial kit (CS0410, Sigma). The conjugation of GSH to 1-chloro-2,4-dinitrobenzene (CDNB) catalyzed by GST was monitored at 340 nm for 4 min (SyneryTM HTX Multi-Mode). The reaction mixture contained 4 μL of extract and 196 μL of reaction solution (200 mM GSH and 100 mM CDNB in Dulbecco’s buffer at pH 7). The activity was calculated with ε = 9.6 mM−1 cm−1 [25]. A GST unit is defined as the amount of enzyme that catalyzes the formation of 1 μmol of the GS-DNB conjugate per minute at 25°C and pH 7. All reagents for oxidative stress detection were analytical grade from Sigma-Aldrich (St. Louis, MO, USA). The analysis were carried out on five subsamples for each site.

### Statistical analysis

One-way ANOVA was used to analyse the differences in metals concentration between sites (in-field experiment), and differences in ROS content and GST activity between treatments in-vitro experiment. The one-way ANOVA was followed by Student-Neuman-Keuls test for post hoc comparisons. Prior to analysis, data not matching a normal distribution (Shapiro–Wilk W test, p <0.05) were log-transformed to correct for skewed distributions. Results were reported as mean ± standard deviation. For the in-field experiment, the relationships between toxic metals content, ROS and GST were assessed using Pearson correlation analysis. Data from all sites (A, B, C) were analysed together.

## Results

### Accumulation of toxic metals

Metal concentrations measured in moss bags exposed at site A (Table 1) are significantly lower than the concentrations measured at sites B and C. In comparison with site A, only Pb significantly (P<0.05) increased at site B and the same occurred at site C compared with site B (Table 1). At site C, Cd, Cu, Cr and Fe concentrations significantly increased compared with sites A and B. Only Ni and Zn concentrations remained significantly unchanged at the three sites (Table 1).

**Table 1:** Concentrations (μg g^-1^ dw) of toxic metals in moss bags of *L*. *riparium* exposed for 7 days at the 3 sites (A, B, C) along the river Sarno.

Elements	Site A	Site B	Site C	*p*
**Cd**	0.11 ± 0.02 a	0.147 ± 0.04 a	0.34 ± 0.04 b	**0.000**
**Cr**	0.26 ± 0.03 a	0.91 ± 0.13 a	5.65 ± 1.19 b	**0.000**
**Cu**	29.1 ± 6 a	29.1 ± 5.9 a	39.2 ± 3.71 b	**0.050**
**Fe**	174.8 ± 2 a	387.7 ± 56.9 a	1715.7 ± 442.4 b	**0.000**
**Ni**	7.9 ± 1.3	7.5 ± 1.9	10.07 ± 4.01	0.425
**Pb**	2.8 ± 0.1 a	3.9 ± 0.05 b	8.4 ± 0.94 c	**0.000**
**Zn**	457.8 ± 133.4	464.2 ± 98.3	467.47 ± 80.9	0.992

Data are shown as the mean ± standard deviation (n = 3) for each site. Values not accompanied by the same letter are significantly different at p < 0.05, using post hoc Student-Neuman-Keuls test. On the right panel, results of one-way ANOVA for each parameter are shown. Significant p-value (p < 0.05) are marked in bold.

### Ultrastructural observations

#### In field

**Fig 1:**
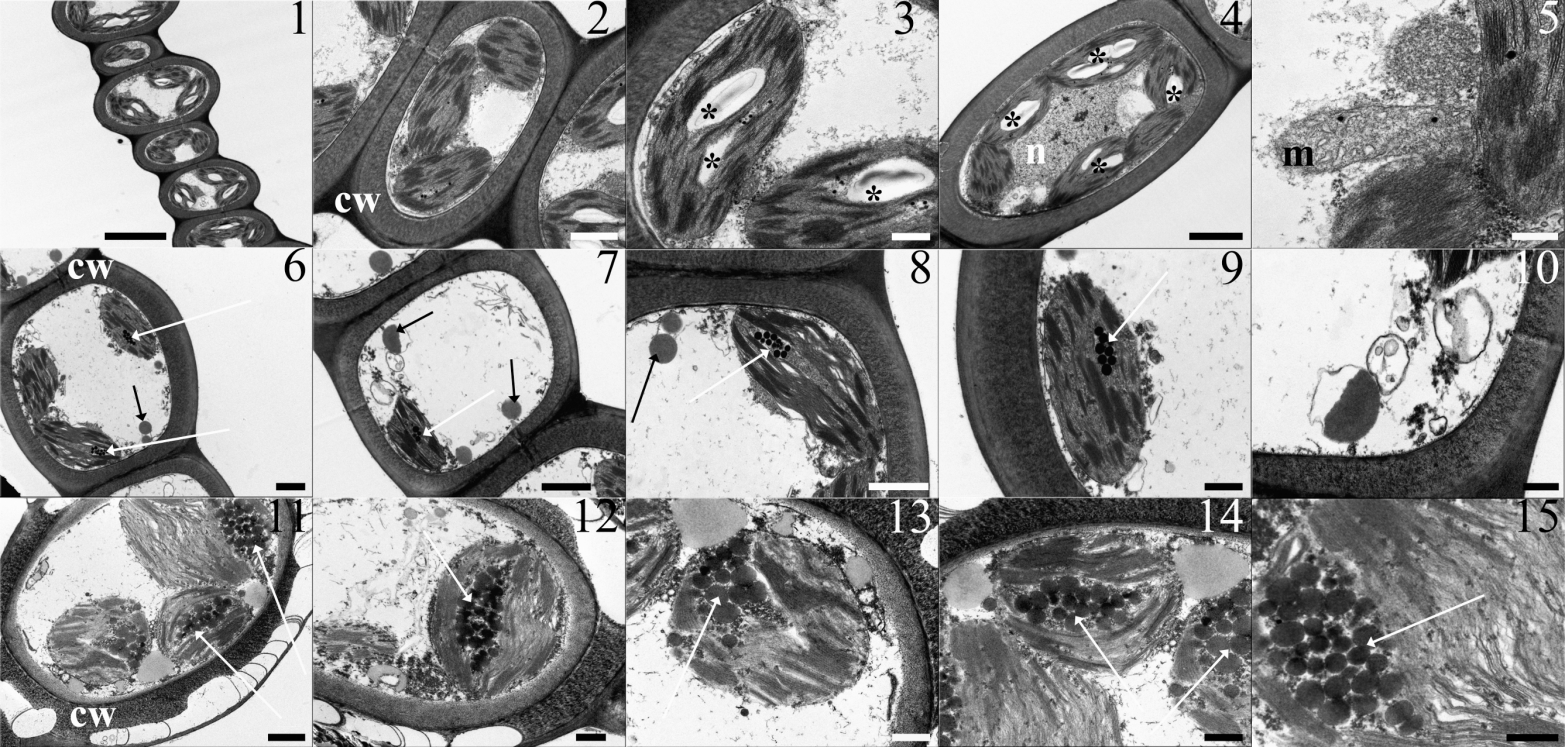
**TEM micrographs from leaflets of *L*. *riparium* specimens exposed in the river Sarno at the site A (1–5), site B (6–10) and site C (11–15).** Site A. (1) Thick wall delimited cells showing lenticular chloroplasts, with grana and starch grains, and large clear vacuoles occupying the centre of the protoplast. (2) A thick walled cell with regular chloroplasts and vacuole. The chloroplasts show well-developed grana. (3) The chloroplasts contain a well-developed thylakoid system, starch grains and rare plastoglobules. (4) The thick wall delimited cell shows regular chloroplasts, with grana and starch grains, and a central nucleus, with eu- and heterochromatin. (5) A section of a mitochondrion with cristae. Site B. (6) A thick wall delimited cell containing chloroplasts, with grana and plastoglobules, and cytoplasm lipid droplets. (7) The cell contains a miss-shaped chloroplast with grana and plastoglobules, cytoplasm lipid droplets and vesicles. (8) A miss-shaped chloroplast with a well-developed thylakoid system and plastoglobules. Lipid droplets and a mitochondrion with no cristae are between the chloroplasts. (9) A chloroplast with a well-developed thylakoid system and plastoglobules. (10) Vesicles at high magnification. Site C. (11) A severely altered cell featured by a highly fissured thick wall. The chloroplasts, still showing a developed thylakoid system with grana, are swollen and filled with large plastoglobules. Large lipid droplets are in the cytoplasm. (12) The altered cell shows cytoplasm lipid droplets and a swollen chloroplast with plastoglobules and thylakoids. (13–14) Chloroplasts showing large plastoglobules and thylakoid systems with still recognizable grana and intergrana membranes. Large lipid droplets around the chloroplast. (15) Magnified plastoglobules and thylakoids. Scala bars: 5 μ (1), 2 μ (4), 1 μ (2, 6, 7, 11, 15), 500 nm (3, 9, 12, 13, 14), 300 nm (5, 10)**. Lettering and marks: cw** cell wall; **m** mitochondrion; **n** nucleus; * starch grain; **black arrow** cytoplasm lipid droplet; **white arrow** plastoglobules.

Moss samples exposed at site A showed an ultrastructure comparable with control, unexposed samples, collected at the Botanical Gardens (data not shown) (Fig 1, Panels 1 to 5). The leaflet cells were delimited by thick cell walls, and contained lenticular chloroplasts beneath the cell wall. The thylakoid system was well developed, and arranged as grana and intergrana thylakoids extending along the longitudinal axis of the organelle: starch grains and rare plastoglobules were visible in the chloroplasts. Large clear vacuoles occupied the centre of the protoplast. Mitochondria with cristae, nucleus with eu- and heterochromatin, endoplasmic reticulum, and dictyosomes were regular.

Samples exposed at sites B and especially C showed severe alterations. After a 7-day exposure at site B, the cells, delimited by a thick cell wall, contained few chloroplasts with respect to those exposed at site A (Fig 1, Panels 6 and 7). These organelles appeared misshaped, but they still preserved grana and intergrana thylakoids; plastoglobules increased (Fig 1, Panels 8 and 9). The cytoplasm showed lipid droplets, vesicles with an electron dense content, and multivesicular bodies (Fig 1, Panels 10). Moss samples exposed at site C were severely damaged: the thick cell walls were highly fissured (Fig 1, Panels 11). The chloroplasts were swollen and contained numerous large plastoglobules; grana were still noticeable (Fig 1, Panels 11 to 15). Large lipid droplets were present in the cytoplasm (Fig 1, Panels 13 and 14).

### In vitro

#### Toxic metals mixture-treatment

**Fig 2:**
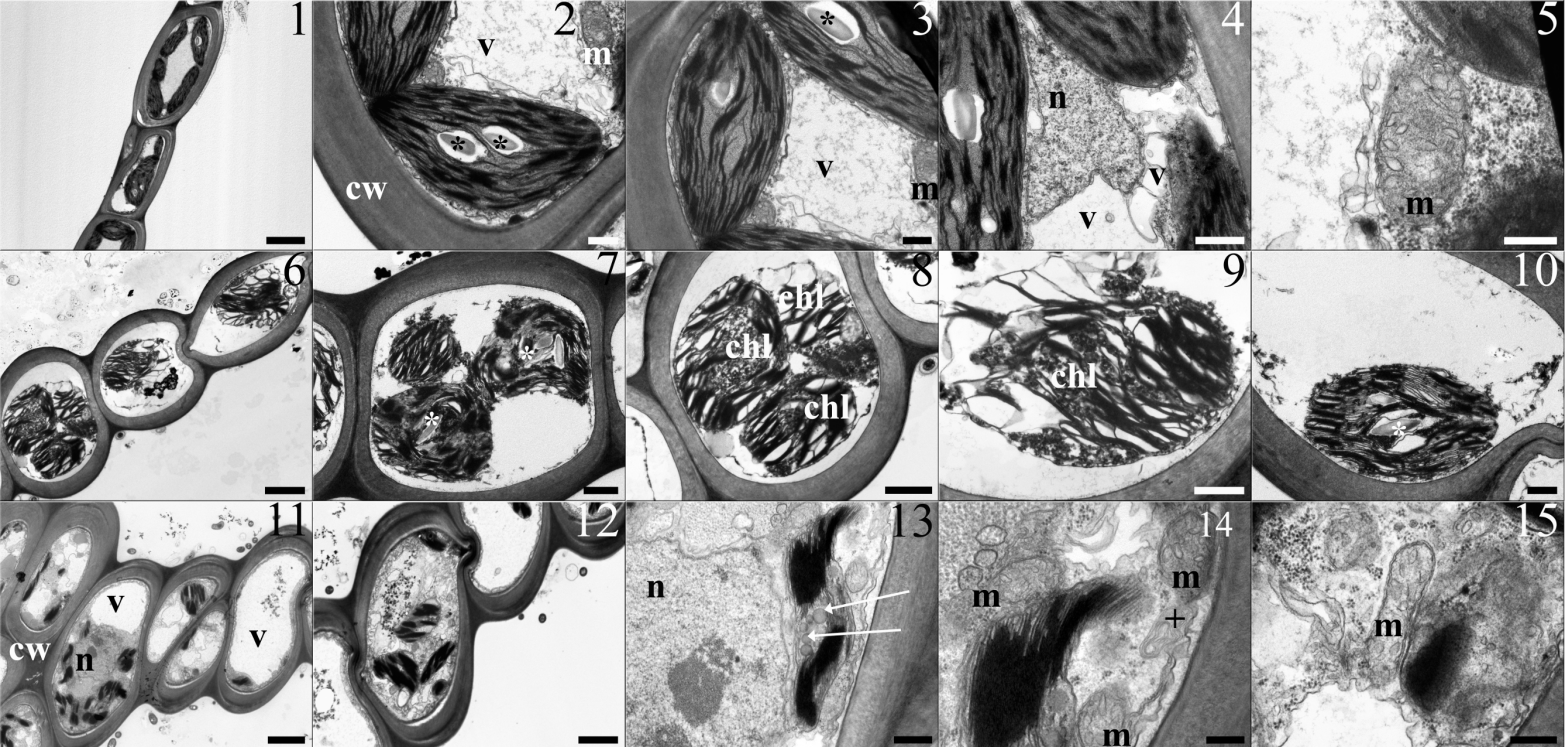
The table shows TEM micrographs from leaflets of *L*. *riparium* specimens cultured in the toxic metal mixture at the same concentrations as in the site A (1–5), site B (6–10) and site C (11–15). Site A. (1) Thick wall delimited cells containing lenticular chloroplasts, with grana and starch grains, and large clear vacuoles. (2, 3) Regular chloroplasts featured by a well-developed thylakoid system and mitochondria. (4) A central nucleus (N) with eu- and heterochromatin, surrounded by chloroplasts and vacuoles. (5) A section of a mitochondrion with cristae. Site B. (6) Thick wall delimited cells showing plasmolysed protoplasts with severely swollen chloroplasts. (7, 8) Severely plasmolysed cells containing swollen chloroplasts with swollen thylakoids. (9, 10) Swollen chloroplasts with swollen thylakoids and small starch grains. Membranes have a thicker and not sharp appearance. Site C. (11) Inside the thick wall delimited cells are changed chloroplasts, vacuoles and a nucleus. (12) A cell with chloroplasts and plenty of cytoplasm vesicles. (13) A detail of a cell showing a misshaped chloroplast with grana and large plastoglobules in the stroma. A nucleus with eu- and etherochromatin is on the left. (14) Details of a misshaped chloroplast with grana, mitochondria and a multilamellar body. (15) A section of a mitochondrion with cristae. Scale bars: 5 μm (1), 3 μm (11), 2 μm (6, 12), 1 μm (4, 7, 8), 500 nm (2, 3, 9, 10, 13, 14), 300 nm (5, 15). Lettering and marks: cw cell wall; m mitochondrion; n nucleus; v vacuole; * starch grain; white arrow plastoglobules; + multilamellar body.

Samples exposed to the toxic metal mixture at the same concentrations as at site A showed the same appearance as control specimens, with no visible ultrastructural damage (Fig 2, Panels 1 to 5). In contrast, samples treated with the toxic metal mixture as at site B appeared severely damaged: heavy plasmolysation, swelling of the chloroplasts and the thylakoids occurred (Fig 2, 6–10). Membranes had a thick appearance. In the samples treated with the toxic metals at the same concentrations as at site C, the chloroplasts were severely misshaped and developed plastoglobules in the stroma; multilamellar bodies occurred in the cytoplasm (Fig 2, Panels 11 to 14). Nuclei and mitochondria were unchanged (Fig 2, Panels 11 to 15).

#### Single toxic metal-treatments

**Fig 3:**
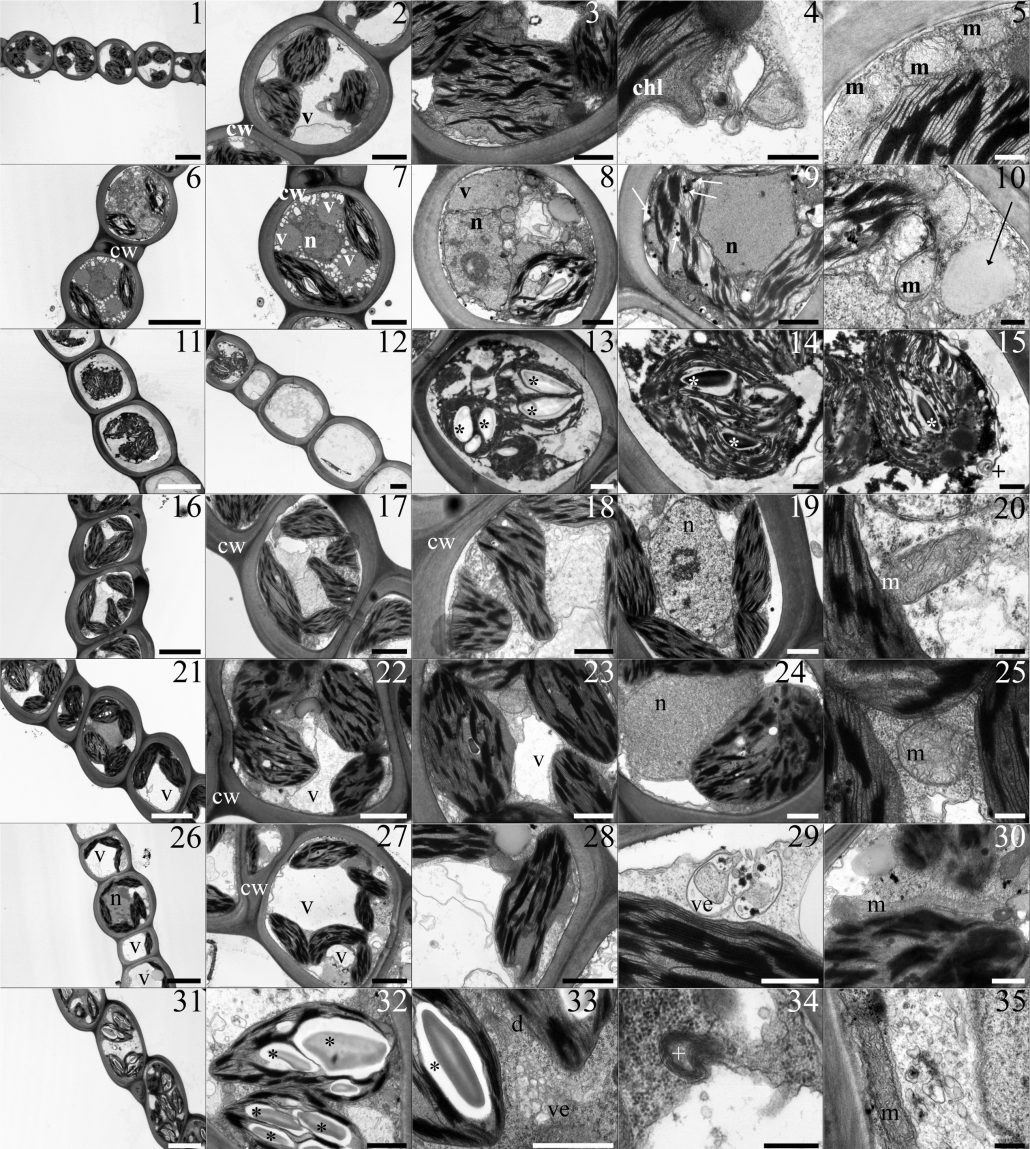
The table shows TEM micrographs from *L*. *riparium* leaflets of samples treated with the single toxic metals. **(1–5) Cd-treated samples.** (1) Thick wall delimited cells with chloroplasts, nucleus and vacuoles. (2) A plasmolysed cell showing a large vacuole and chloroplasts with grana. (3) A misshaped chloroplast with grana and integrana thylakoids. (4) A bulge from a misshaped chloroplast. (5) Beside a chloroplast mitochondria with cristae. **(6–10) Cr-treated samples.** (6) Thick wall delimited cells with highly vacuolated cytoplasm, chloroplasts and nuclei. (7) The cell cytoplasm contains clear and electron dense vacuoles, chloroplasts and a nucleus. (8) The plasmolysed cell presents a misshaped chloroplast, with grana and a starch grain, clear and electron dense vacuoles, a nucleus and a large lipid droplet in the cytoplasm. (9) The cell contains a central nucleus surrounded by misshaped chloroplasts with a poorly developed thylakoid system and visible plastoglobules. (10) A diving mitochondrion, with clear matrix and no cristae, located beside a cytoplasm lipid droplet. **(11–15) Cu-treated sample.** (11–12) Severely plasmolysed cells with swollen chloroplasts and empty cells are shown. (13) A plasmolysed cell filled with swollen chloroplasts provided by starch grains. (14–15) Plasmolysed cells containing swollen chloroplasts with visible grana and starch grains. A multilamellar body near the plasma membrane. All the membranes appear poorly sharp. **(16–20) Fe-treated samples.** (16–18) Thick wall delimited cells with misshaped chloroplasts containing grana. (19) A normal nucleus with eu- and heterochromatin and a nucleolus. (20) A section of a mitochondrion with cristae. **(21–25) Ni-treated samples.** (21) The thick wall delimited cells show chloroplasts with grana and clear vacuoles. (22–23) Cells containing clear vacuoles and chloroplasts with grana. (24) A nucleus next to a chloroplast. (25) A section of a mitochondrion with developed cristae. **(26–30) Pb-treated samples.** (26) Thick wall delimited cells with chloroplasts and large clear vacuoles. (27) A cell contains chloroplasts with grana and clear vacuoles. Some of the chloroplasts appear misshaped. (28) A misshaped chloroplast with grana. (29) A vesicle filled with material fuses with plasma membrane. (30) A longitudinal section of a mitochondrion with cristae **(31–35) Zn-treated samples.** (31) Cells with chloroplasts containing starch grains. (32) A chloroplast with grana and starch grains. (33) Plenty of vesicles in the cytoplasm beside dictyosomes and chloroplasts. (34) A multilamellar body. (35) A longitudinal section of a mitochondrion with cristae, next to cytoplasm vesicles. **Scale bars: 5** μ**m** (1, 6, 11, 16, 21, 26, 31), **2** μ**m** (2, 7, 12, 17, 22, 27), **1** μ**m** (3, 8, 9, 13, 18, 19, 23, 24, 28, 32, 33), **500 nm** (4, 14, 15, 29, 30), **300 nm** (5, 10, 20, 25, 34, 35)**. Lettering and marks: chl** chloroplast; **cw** cell wall; **d** dictyosomes; **m** mitochondrion; **n** nucleus; **v** vacuole; **ve** vesicles; * starch grain; **white arrow** plastoglobules; **+** multilamellar body.

Cd-treated samples. Cd-treated samples maintained a regular ultrastructure arrangement (Fig 3, Panels 1). Leaflet cells showed chloroplasts with a well-developed thylakoid system (Fig 3, Panels 2). Chloroplasts appeared misshaped as their profile was wavy, with bulges and invaginations (Fig 3, Panels 3 and 4). Nuclei and mitochondria were comparable to the control (Fig 3, Panels 5).

Cr-treated samples. Cr-treatment induced cytoplasm vacuolization with the occurrence of both clear and electron dense vacuoles (Fig 3, Panels 6 and 8). Chloroplasts, even though still maintaining a regular grana and intergrana arrangement, showed irregular profiles (Fig 3, Panels 8 and 9). Mitochondria with clear matrix and very few cristae, and cytoplasmic lipid droplets occurred (Fig 3, Panels 10).

Cu-treated samples. Cu-treatment induced changes of the ultrastructure. The leaflet cells appeared heavily plasmolysed, or even empty (Fig 3, Panels 11 and 12). Chloroplasts were swollen, even though grana and intergrana thylakoids and starch grains were still visible (Fig 3, Panels 13 and 14). Membranes had a thick appearance. Multilamellar bodies occurred in the cytoplasm (Fig 3, Panels 15).

Fe-treated samples. Fe-treated samples maintained the ultrastructure arrangement, but chloroplast shape was changed (Fig 3, Panels 16 to 18). Nuclei and mitochondria were comparable to the control (Fig 3, Panels 19 and 20).

Ni-treated samples. In Ni-treated samples, chloroplasts still maintained grana and intergrana arrangement (Fig 3, Panels 21 to 24). Electron dense and electro clear vacuoles were observed. (Fig 3, Panels 21 to 24). Other organelles such as mitochondria still preserved their regular ultrastructure (Fig 3, Panels 25).

Pb-treated samples. Pb treatment induced chloroplast misshaping (Fig 3, Panels 26 to 28); a swelling of the space between the outer and inner membranes occurred (Fig 3, Panels 28). Furthermore, cytoplasm showed vesicles filled with electron dense material (Fig 3, Panels 29). The other organelles were comparable with the control (Fig 3, Panels 30).

Zn-treated samples. Zn treatment induced the presence of cytoplasm vesicles, and multilamellar bodies (Fig 3, Panels 34 and 35). Chloroplasts conserved a well-developed thylakoid system and starch grains (Fig 3, Panels 31 to 32); no other changes were observed in the other organelles (Fig 3, Panels 35).

### Oxidative stress

#### In field

**Fig 4:**
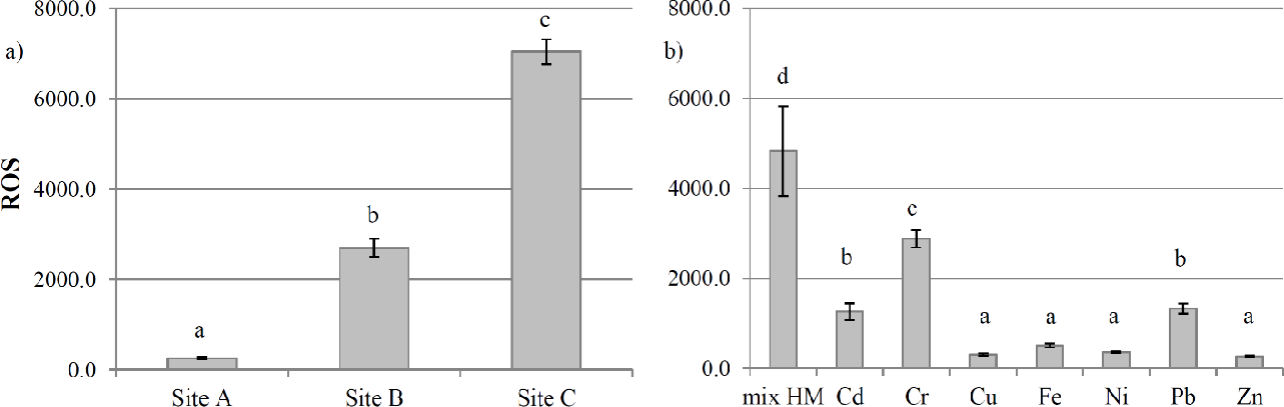
**ROS content in *L*. *riparium* exposed in bags at sites A, B and C of Sarno River (in-field experiment, left panel, a) and *in vitro* cultured with mixtures of toxic metal or with the single toxic metal at the concentrations measured in site C (CdCl**_**2**_ **0.14 mg l**^**-1**^**, Cr(Cl) 3 9.05 mg l**^**-1**^**, CuSO**_**4**_ **2.45 mg l**^**-1**^**, FeCl**_**2**_ **308.0 mg l**^**-1**^**, NiCl**_**2**_ **3.4 mg l**^**-1**^**, Pb(CH3COO)**_**2**_ **0.85 mg l**^**-1**^**, ZnCl**_**2**_ **46.76 mg l**^**-1**^**) (right panel, b).** Data are shown as the mean ± standard deviation (n = 5). The ROS quantity was monitored by fluorescence (excitation wavelength of 350 nm and emission wavelength of 600 nm). Bars not accompanied by the same letter are significantly different at p < 0.05, using post hoc Student-Neuman-Keuls test.

**Fig 5:**
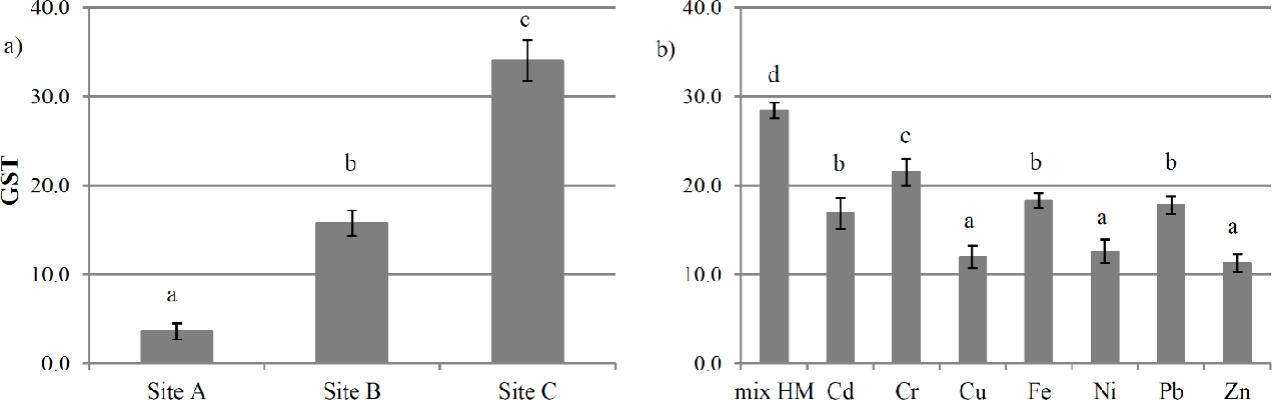
GST activity in *L*. *riparium* exposed in bags and *in vitro* cultured. GST activity in *L*. *riparium* exposed in bags at sites A, B and C of Sarno River (in-field experiment, left panel, a) and *in vitro* cultured with mixtures of toxic metal or with the single toxic metal at the concentrations measured in site C (CdCl_2_ 0.14 mg l^-1^, Cr(Cl) 3 9.05 mg l^-1^, CuSO_4_ 2.45 mg l^-1^, FeCl_2_ 308.0 mg l^-1^, NiCl_2_ 3.4 mg l^-1^, Pb(CH_3_COO)_2_ 0.85 mg l^-1^, ZnCl_2_ 46.76 mg l^-1^) (right panel, b). Data are shown as the mean ± standard deviation (n = 5). The GST activity was expressed as micromoles/ml min^-1^. Bars not accompanied by the same letter are significantly different at p < 0.05, using post hoc Student-Neuman-Keuls test.

ROS levels were low in *L*. *riparium* field-exposed at site A, but they significantly increased from site B to site C, by 9.4- and 26-fold respectively, compared with site A (Fig 4A). GST activity in field-exposed samples increased significantly from site B to site C, reaching the maximum at site C (Fig 5A).

#### In vitro

The treatment with the metal mix as measured at sites A and B did not show significant differences in ROS concentrations and GST activity measured in field-exposed samples (data not shown). *In vitro* treatment with the same metal mixture as site C gave a lower ROS value than field-exposure at the corresponding site, with a 31% lower value. Among the toxic metals tested, using concentrations as at site C, Pb, Cr and Cd caused the highest increase in ROS content, by 4-, 10-, and 4-fold respectively, compared with site A (Fig 4B). Treatment with the same metal mixture as at site C increased GST activity 7-fold, compared with the mixture as at site A (data not shown). As for the single metal treatments, similarly to ROS, treatments with Pb, Cr and Cd showed the maximum GST activities (Fig 5B).

### HSP70s induction

#### In field

**Fig 6:**
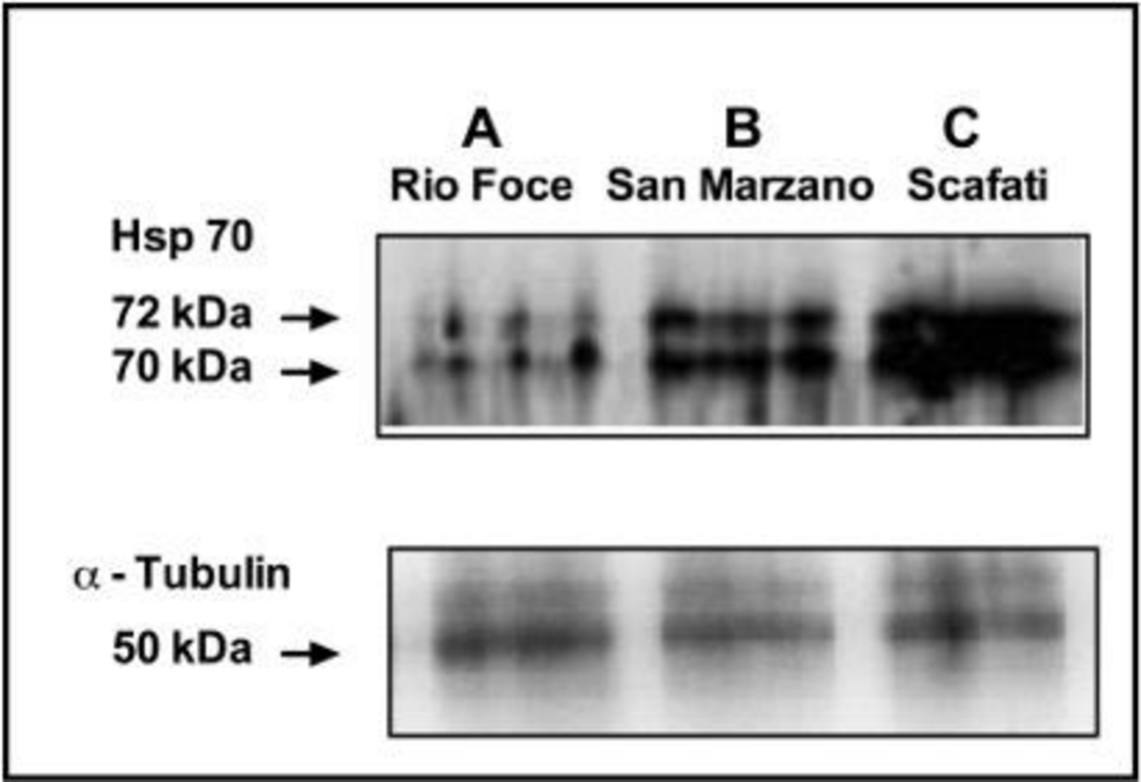
**Western blotting using Hsp70 antibodies (Sigma) of *L*.*riparium* samples exposed along the Sarno River at sites A, B and C.** The lower figure shows Western Blotting of the same samples using alfa-tubulin to check the equal loading of electrophoretic lanes.

Moss samples of *L*. *riparium* exposed in bags along the Sarno River for 7 d showed strong differences in the amount of proteins reacting vs HSP70 antisera (Fig 6). At site A two pale bands of proteins were detected. At site B a strong increase was observed in two bands at 72 and 70 kDa MW. These bands further increased at site C, confirming the high and increasing degree of water pollution along the river.

#### In vitro

**Fig 7:**
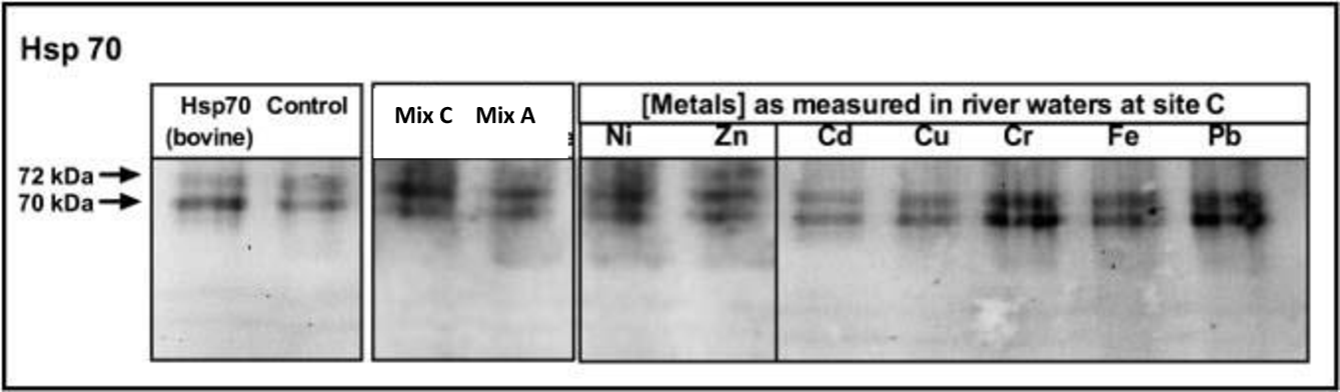
Western blotting using Hsp70 antibodies (Sigma) of *L*. *riparium* samples exposed *in vitro*. Western blotting using Hsp70 antibodies (Sigma) of *L*. *riparium* samples exposed *in vitro* to metal mixtures of site C and site A, and to the single metal concentrations as measured in the site C: CdCl_2_ 0.14 mg l^-1^, Cr(Cl) 3 9.05 mg l^-1^, CuSO_4_ 2.45 mg l^-1^, FeCl_2_ 308.0 mg l^-1^, NiCl_2_ 3.4 mg l^-1^, Pb(CH_3_COO)_2_ 0.85 mg l^-1^, ZnCl_2_ 46.76 mg l^-1^.

The Fig 7 shows that HSP70s also increased exposing *L*. *riparium in vitro* to the toxic metals: the most severe increase was induced by the metal mixture as at site C and by Pb, Cr and Ni, when tested as single metals.

#### Pearson’s correlation

In Table 2, data collected for in-field experiment were pooled together and the correlation between heavy metals and ROS and GST were derived. The amount of ROS, proportional to the oxidative stress experienced in the sample, resulted directly correlated with the concentration of all metals measured, except for Ni and Zn. In agreement with this result, the antioxidant activity was not related with the concentration of Ni and Zn. All metals were directly intercorrelated (i.e. Cd increases with increasing of Cr), with the exception of Ni and Zn.

**Table 2:** Pearson correlation coefficients (r) obtained for the linear correlations between GST and ROS and metals concentrations measured in-field experiment.

	GST	ROS	Cd	Cr	Cu	Fe	Ni	Pb	Zn
**GST**	1	**0.989**	**0.889**	**0.935**	**0.616**	**0.927**	0.412	**0.956**	0.024
**ROS**		1	**0.928**	**0.934**	**0.642**	**0.920**	0.420	**0.971**	0.030
**Cd**			1	**0.951**	**0.685**	**0.942**	0.529	**0.937**	-0.01
**Cr**				1	**0.738**	**0.995**	0.473	**0.943**	0.087
**Cu**					1	**0.733**	0.337	**0.657**	**0.656**
**Fe**						1	0.540	**0.937**	0.062
**Ni**							1	0.545	-0.27
**Pb**								1	-0.03
**Zn**									1

Data from all experimental sites were analyzed together. Correlations with p< 0.05 are marked in bold.

## Discussion

The use of living organisms to survey water ecosystems has been recommended by the European Union (Water Framework Directive, 2000/60/CE), and aquatic mosses are known to be suitable bioaccumulators of trace elements [26]. Mosses react to toxic metals excess in a complex way, activating a number of pathways at physiological and morphological levels and in the present work both have been studied and related to bioaccumulation shown both in the field and in vitro experiments.

The Sarno River, one of the most polluted in Europe, is known for having been subjected to illegal disposal of waste from the leather industry [27]. Toxic metals concentrations measured in moss bags exposed at site A (Tab. 1) were generally in line with values reported for aquatic mosses from unpolluted or lightly polluted rivers [28, 29]. This confirmed that site A, at the springs of the Sarno River, is not affected by toxic metal pollution. On the contrary moss bags bioaccumulation in site B and C confirmed the high degree of pollution of these sites, pinpointing at toxic metals as polluting the waters of the Sarno River. The pollution source is connected with the widespread presence of leather factories, massively using Cr and Fe for tanning, and the possible illegal waste disposal. Nowadays, Cr is largely replaced by Al in tanning manufacturing. At sites B and C the concentrations of Cd, Cu, and Pb were high as well, claiming for environmental and human health concern, and suggesting that the use of untreated river waters, e.g. for irrigation of agricultural products, should be avoided to prevent toxic metal accumulation along the food chain, with possible effects on human health. The ultrastructural observations of samples cultured *in vitro* and exposed to the toxic metal mixture, confirmed that low toxic metal concentrations as measured at the site A did not induce ultrastructure damage [19]. On the contrary, also from *in vitro* data we can conclude that the toxic metal concentrations measured at sites B and C cause severe and increasing alterations; this is consistent with our previous studies [6,7,19,22]. In addition, it should be considered that not all the metals, or not all fractions, present in the river waters may be readily available to the moss, because toxic metals could be not available to biological organisms, and thus not harmful [30,31]. The use for the *in vitro* test of fully soluble salts could also explain the more visible plasmolysis in the *in vitro*-treated samples compared with the in field-exposed ones. Intriguingly, samples treated with the metal mixture similar to the water of site B are all plasmolysed, but samples exposed at site B were not: this could be possibly caused by a different forms and bioavailability of pollutants. This hypothesis has been supported by Sassmann et al. [32], who found free metal ion availability as a major factor for tolerance and growth of plant under metal treatments.

Culturing *in vitro* with single toxic metals induced harmful effects on the moss, resulting in plasmolysis, swollen chloroplasts, and thick membrane appearance; moreover, empty cells appeared along the leaflet section. Toxic metals induced harmful effects, possibly in relation to their toxicity, concentration and/or availability.

The ultrastructural analyses of *L*. *riparium* cells showed that the most damaged organelles were the chloroplasts. In our samples these organelles became heavily swollen and developed large plastoglobules in the stroma. Chloroplasts are a common target of metal toxicity in different plant taxa, as comparable damage was already reported for toxic metal-treated *L*. *riparium* [5], and other bryophytes, like the mosses *Funaria hygrometrica* (Hedw.) [33] and *Scorpiurum circinatum* (Brid) [34] the liverworts *Pellia neesiana* (Gottsche) Limpr. [35] and *Lunularia cruciata* L. Dum [36], as well as the aquatic Angiosperms *L*. *minor* and *Elodea canadensis* Michx. [19, 37].

Toxic metal exposure is known to induce oxidative stress [30] and lipid peroxidation [38]; thus, cell membranes and membrane-rich organelles, such as chloroplasts, and their functionality, such as selective permeability, are expected targets of harmful effects. All that could also cause swelling or shrinkage of the whole cell or its organelles, observed in our treated samples, relating to membrane impairment.

Damaged membranes could be the source for the lipid droplets and plastoglobules [38–40], which increase in our treated samples. In plastoglobules these lipids could be recycled for the synthesis of tocopherols and vitamin E [41], which are also able to scavenge ROS [42]. Metal-induced toxicity and oxidative stress could also explain the occurrence of multivesicular bodies. These ultrastructures, which were observed in our treated samples, originate as an accretion of undigested membranes from endocytosis phenomena, probably related to autophagy recycling damaged cell components [43, 44].

Plant vacuoles, which are also increased in some of our treated samples, are also known to be involved in autophagy phenomena and are reported to be a major site for the degradation of macromolecules [42, 45].

*L*. *riparium* samples exposed along the Sarno river path showed strong GST activity, increasing from site A to site C. This can enable the cells to better scavenge the pollution-induced ROS increase. Therefore, it can be suggested that plant adaptive response(s) to pollution-induced oxidative stress may involve antioxidants like GST. The correlation between GST activity and ROS levels implies that the induction of activity of this enzyme by pollution is attributable to enhanced ROS. Therefore, activation of glutathione-S-transferases could enhance ROS quenching.

Heavy metals stimulated the GST activity. Notably this effect seems not being species-specific, since it has been found in several studies, also on pumpkin (*Curbita maxima*) subjected to metal stress [46, 47] and in rice in response to Cd stress [48].

Antioxidant activities linearly and progressively increased along the Sarno river path; in contrast, antioxidant activities observed in *in vitro* metal-treated samples showed a lower increase. This suggests that the antioxidant activity in the field-exposed samples could be inducted by others, but not yet measured, pollutants as well. This is in agreement with the trend of the ultrastructure damage revealed by our TEM observations, and HSP70s induction. *L*. *riparium* GST activity trend along the Sarno river suggests that many sources of pollution are present along the river path [49, 50].

On the other hand, the correlation between metal pollution and ROS/GST levels is very strong (>92%).

Increased levels of HSP70s are usually resulting from the toxic action of pollutants taken up by living organisms, which have not been scavenged by detoxification systems, and negatively influence the correct folding of native proteins [51]. The moss *L*. *riparium* increased the activity of HSP70s in parallel with a higher accumulation of toxic metals. This result is consistent with the increased HSP70s and antioxidant activity found in experiments with the same moss exposed *in vitro* to toxic metals [5]. It is assumed that this response could be induced by toxic metal stress and has a protective role against toxic effects.

A previous experiment was carried out in Sarno River using the aquatic macrophyta *Lemna minor* L. [19] and a comparable pollution was detected. In that case both ultrastructure and HSPs responded consistently with the metallic concentrations measured in the same 3 sites. Compared to *L minor*, *L*. *riparium*, although presenting a simpler anatomical organization, typical of mosses, presents a surprising more preserved ultrastructure. In particular, the thylakoid system still appears recognizable even in the samples exposed in the most polluted site and /or at the highest metal concentrations. In addition, the measured bioaccumulation confirms that the moss is not able to avoid the presence of metals, but rather it accumulates them. The relative "resistance" of the moss is explained by the biochemical responses measured. In particular both HSPs and antioxidant response could protect the moss from proteotoxic and oxidative stresses respectively. The observed strong responses of *L riparium* could explain its hard resistance to pollutants and suggest it as an excellent bioindicator of pollution in aquatic systems.

In conclusion, for the first time to our knowledge, a comprehensive study has described the effects of pollution in moss in a river, by comparing both ultrastructural damage with biochemical and physiological parameters observed *in situ* and carefully measuring pollution in river waters. Always for the first time, these alterations have been validated by comparing the response in field with the parameters measured in the laboratory under controlled conditions. Overall, our results suggest that *L*. *riparium* is able to respond to pollution by modifying biological parameters, such as HSP70s, ultrastructural organization, enzyme and antioxidant activity, suggesting that this species may be used as an effective bioindicator and bioaccumulator of water toxic metal pollution.

## Supporting information

S1 Table Concentrations of metals as mg l^-1^ in waters of Sarno River, measured at the three different exposure sites. (TIFF)
