# Supplementary figures and images for: In-field and in-vitro study of the moss Leptodictyum riparium as bioindicator of toxic metal pollution in the aquatic environment: Ultrastructural damage, oxidative stress and HSP70 induction
Source: PLoS One. 2018 Apr 12;13(4):e0195717. doi: 10.1371/journal.pone.0195717 (PMC5896978; doi:10.1371/journal.pone.0195717)

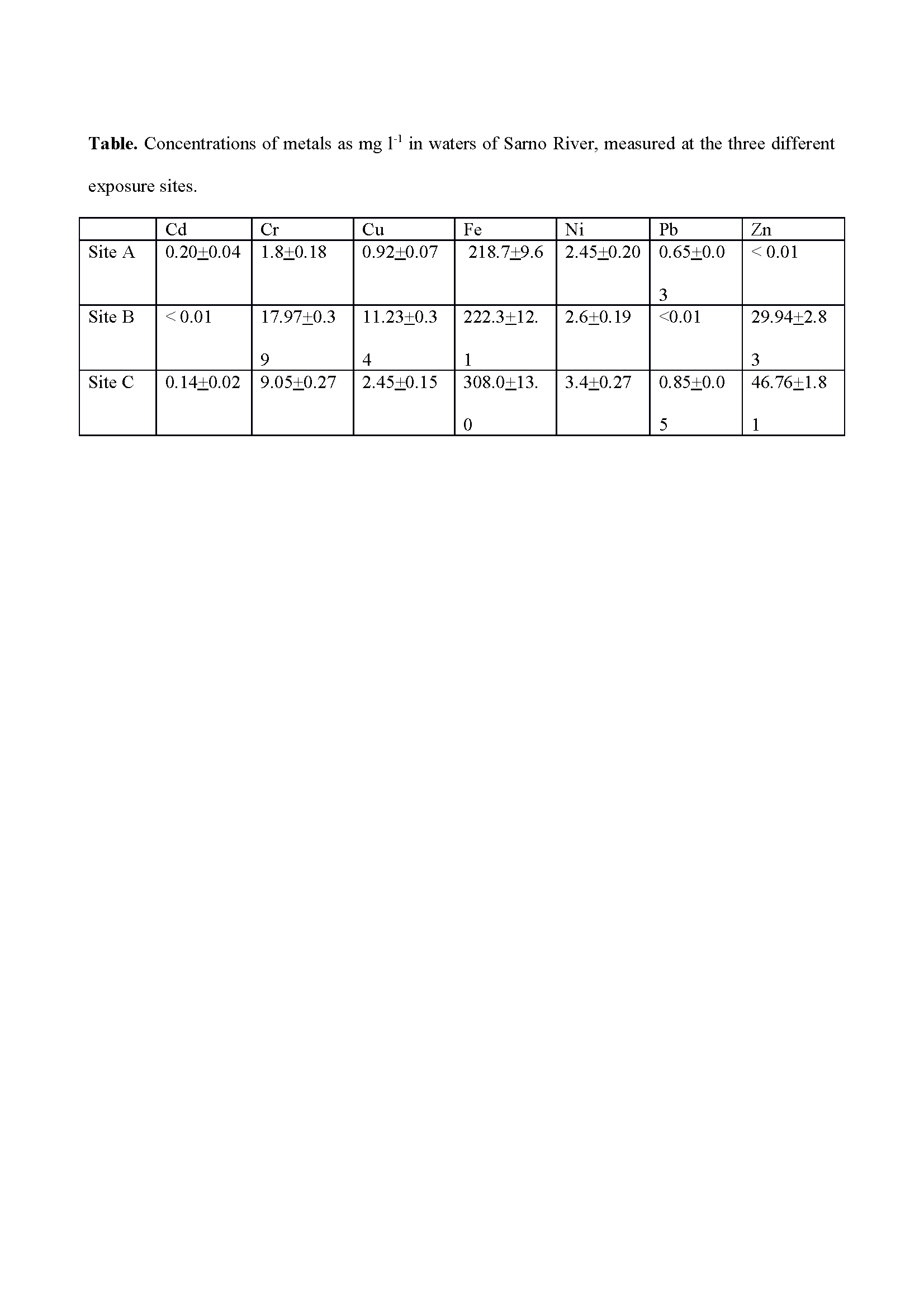

Supplement: S1 Table — (TIFF) [file pone.0195717.s001.tiff]
